# Impact of minimal self disorders on naturalistic episodic memory in first-episode psychosis and parallels in healthy individuals with schizotypal traits

**DOI:** 10.3389/fpsyt.2024.1469390

**Published:** 2024-11-13

**Authors:** Delphine Yeh, Sylvain Penaud, Alexandre Gaston-Bellegarde, Linda Scoriels, Marie-Odile Krebs, Pascale Piolino

**Affiliations:** ^1^ Université Paris Cité, Laboratoire Mémoire, Cerveau et Cognition, Boulogne-Billancourt, France; ^2^ Université Paris Cité, Laboratoire de Psychologie du Développement et de l’Éducation de l’Enfant, CNRS, Paris, France; ^3^ GHU Paris Psychiatrie et Neurosciences, Paris, France; ^4^ Université Paris Cité, INSERM, Institute of Psychiatry and Neuroscience of Paris, Paris, France

**Keywords:** minimal self, bodily self-consciousness, embodiment, episodic memory, autobiographical memory, first-episode psychosis, schizotypy, virtual reality

## Abstract

**Background:**

Self-disorders constitute a core feature of the schizophrenia spectrum, including early stages such as first-episode psychosis (FEP). These disorders impact the minimal Self, or bodily self-consciousness, which refers to the basic, pre-reflective sense of embodied experience. The minimal Self is intrinsically linked to episodic memory, which captures specific past experiences of the Self. However, research on this relationship in the schizophrenia spectrum remains scarce. This pilot study aimed to investigate how the minimal Self modulated episodic memory of naturalistic events in FEP, using immersive virtual reality. A secondary objective was to examine the relationships between sense of Self, embodiment, episodic memory, schizotypal personality traits in healthy participants (CTL), and psychopathology in FEP.

**Methods:**

A full-body illusion was induced in 10 FEP and 35 matched CTL, using a first-person avatar, with synchronous or asynchronous visuomotor stimulation (strong or weak embodiment conditions, respectively). Following embodiment induction, participants navigated a virtual city and encountered naturalistic daily life events, which were incidentally encoded. Episodic memory of these events was assessed through a comprehensive recognition task (factual and contextual information, retrieval phenomenology). Sense of Self, schizotypal personality traits, and psychopathology were assessed via self-reported questionnaires or clinical assessments.

**Results:**

Synchronous visuomotor stimulation successfully induced a stronger sense of embodiment in both FEP and CTL. The strong embodiment condition was associated with reduced perceived virtual space occupation by the body in FEP. Under strong embodiment, FEP performed significantly worse than CTL in contextual information recognition, but their ratings for retrieval phenomenology were comparable to CTL. Conversely, under weak embodiment, FEP performed similarly to CTL in contextual information recognition, but they rated retrieval phenomenology significantly lower. For CTL, we observed a slight, though non-significant, enhancement in recognition memory under strong compared to weak embodiment. Additionally, higher schizotypy in CTL correlated with a diminished sense of Self and poorer episodic memory.

**Conclusions:**

Disturbances in the minimal Self in FEP are associated with episodic memory impairments. These findings emphasise the importance of targeting minimal Self-disorders in schizophrenia spectrum disorders, since episodic memory impairments may negatively affect patients’ quality of life and psychosocial outcomes. Additionally, they support a fully dimensional model of schizotypy.

## 1 Introduction

The sense of Self refers to the mental processes that enable each individual to define themselves as a unique, coherent, and distinct human being over time (1). The Self is multifaceted, and numerous models have been proposed for its conceptualisation. Two main facets appear to emerge from the various models, although their definitions and scope may vary slightly depending on the authors (1–7). On the one hand, the narrative facet of the Self corresponds to the ‘Me’ object of a reflective mental representation of the self-concept; on the other hand, the minimal facet of the Self refers to bodily self-consciousness, which corresponds to the basic sense of self-awareness that an individual possesses. It describes the fundamental aspects of consciousness and subjective experience and encompasses the ‘I’ subject of the pre-reflective phenomenological experience of being an agent embodied in a body, which constitutes the point of origin of the experience of the world. More specifically, the minimal Self can be divided into four main subcomponents (8), namely: the feeling that my body belongs to me, i.e. body ownership; that I am spatially anchored within it, i.e. self-location; that I perceive the world from this body, i.e. first-person perspective; and that I control the actions of this body, i.e. agency. The minimal Self emerges from the multimodal integration of multisensory, interoceptive, and motor bodily cues (8) within the peripersonal space. The peripersonal space refers to the area immediately surrounding the body and anchored to the body, delineating the space of the Self and mediating the interactions between the body and the environment (9, 10).

Self-disorders have been considered a fundamental characteristic of schizophrenia since its early descriptions (11–13). However, they have been overshadowed by the focus on psychotic symptoms (i.e., delusions, hallucinations, or disorganised speech and behaviour) targeted by antipsychotics, and the challenge of assessing patients’ subjective self-experience reliably. In the early 2000s, self-disorders were re-emphasised as a core psychopathological feature of schizophrenia, due to the development of psychometric instruments such as the Examination of Anomalous Self-Experience (EASE) scale (14–16). These self-disorders affect both the minimal Self (generalised impairments in all subcomponents) (16–20) and the narrative Self (metacognitive impairments and poor insight, i.e. poor awareness of the disease and associated symptoms and consequences) (21–24), and distinguish schizophrenia from other mental disorders (25). Self-disorders are present from the early stages of the schizophrenia spectrum, including the first-episode psychosis (FEP), which corresponds to the first manifestations of psychotic symptoms at levels above the diagnostic threshold. These self-disorders can predict conversion to schizophrenia (25), emphasising the necessity of early intervention (26). Furthermore, self-disorders have been found in schizotypy (27), which is a multidimensional personality trait observed in the general population. Schizotypy lies on a continuum with schizophrenia spectrum disorders and exhibit a dimensional relationship with schizophrenia (28). While some FEP convert to other disorders, self-disorders are more prevalent in schizophrenia spectrum psychosis (29), highlighting their significance for improving diagnosis and treatment in FEP.

Self-disorders have been demonstrated to be associated with the psychopathological dimensions of schizophrenia, i.e. positive, negative, and disorganisation symptoms, as well as impaired social functioning and suicidality (25). Nevertheless, few studies have addressed the impact of self-disorders on episodic memory in the schizophrenia spectrum. This is despite the fact that episodic memory is also impaired in this condition, along with other cognitive deficits (30, 31), and that the relationship between the Self and episodic memory is particularly strong. Indeed, episodic memory refers to the memory of past experiences of the Self and contributes to one’s sense of identity and temporal continuity through autonoetic consciousness, which is a form of self-consciousness enabling one to mentally travel through one’s personal subjective time (1, 32–34). Episodic memory is defined as the memory of specific events that have been personally experienced, are unique, located in time and space, and are associated with a feeling of mentally re-experiencing the past experiences of the Self with a feeling of recollection, combined with specific phenomenological details and a personal perspective of the person who is remembering (32, 33). It is analogous to episodic autobiographical memory, which is constituted by the personal, real-life, specific events that are included in the construction of one’s Self and one’s life story (35, 36).

One classical paradigm that has been used to study the relationship between the Self and episodic memory is the ‘self-reference effect’ (SRE), which posits that processing information closely related to the Self constitutes the optimal strategy for memorising new material (37, 38). The Self thus acts as a processing bias, determining how and which information is encoded and retrieved in memory (1, 34). The SRE has been extensively studied in a laboratory context, with a particular focus on the narrative facet of the Self (39–42). More recently, SRE studies have also begun to focus on the minimal facet of the Self, particularly in light of the advancements in virtual reality technology, which can be defined as the simulation of interactive and multisensory three-dimensional entities in real time to create an immersive experience for the user (43). Indeed, the strong sense of presence created by virtual reality has facilitated the engagement of the Self in an embodied approach to memory, which considers the person’s interactions with their physical bodies and the external world (44–47). The virtual avatar becomes a digital alter ego of the person’s physical Self, located in an embodied virtual environment (48). A study has demonstrated, for instance, that objects encoded in the presence of a visible body in the encoding scene are better recognised than those encoded without a visible body (49). A full-body illusion has been developed to induce the illusion of embodying a virtual avatar, thereby enabling an experimental manipulation of the minimal Self (50, 51). In this illusion, the induction of embodiment in a virtual avatar is achieved through synchronous visuotactile or visuomotor stimulation, whereas the alteration of embodiment is achieved through asynchronous multimodal stimulation. Indeed, the full-body illusion relies on the necessary spatiotemporal synchrony and coherence of perceived bodily cues to ensure the proper functioning of the multimodal integration mechanisms that contribute to the minimal Self (8). Therefore, being embodied from a first-person perspective in a realistic and synchronous avatar, that we identify as our own, mirrors the standard way of experiencing reality for healthy people.

However, classical SRE studies involved assessments of episodic memory that relied on simplistic items with a limited ecological validity, such as lists of words or objects devoid of context. Furthermore, these studies did not consider the expression of episodic memory in everyday life. Consequently, virtual reality has also emerged as a promising approach to assess episodic memory in more naturalistic paradigms, as it can provide immersive, interactive, and multisensory scenes while allowing for control and standardisation of the experience, which can be challenging to ensure in a natural environment (46, 52). Consequently, the use of virtual reality to both present naturalistic life scenes for the encoding in episodic memory and to manipulate the sense of embodiment has yielded, for instance, evidence of better episodic recollection of naturalistic life scenes encoded with a first-person perspective in comparison to encoding with a third-person perspective (53). Other studies have employed the synchrony or asynchrony of visuotactile stimulations to induce a strong or weak sense of embodiment in a virtual avatar. These studies have demonstrated that, in comparison to encoding life scenes when weakly embodied (asynchronous visuotactile stimulation), encoding when strongly embodied (synchronous visuotactile stimulation) results in better immediate accuracy and recall, as well as better accuracy and greater phenomenological experience after one week (54). However, none of these studies proposed a comprehensive episodic memory test that assessed all facets of episodic memory. These facets include memory accuracy, recognition sensitivity, spatiotemporal and source information, sense of recollection, phenomenology of retrieval, and memory binding. Memory binding is a core cognitive process of episodic memory by which different components of a memory are integrated and stored as a coherent whole, enabling individuals to recall complex memories as unified experiences rather than disjointed fragments (55). Therefore, in a previous study conducted with healthy participants, we used visuomotor stimulation to induce embodiment in an avatar and conducted a comprehensive assessment of the episodic memory of naturalistic events using a standardised episodic memory task developed by our team in recent years. The task was based on the encoding of daily life scenes encountered while walking in a virtual city using either non-immersive or immersive virtual reality (44, 45). We found that recall, memory for details, and associative memory for factual and contextual elements of the scenes encoded when strongly embodied (synchronous visuomotor stimulation) were superior to those encoded when weakly embodied (asynchronous visuomotor stimulation) in healthy participants (45).

In the schizophrenia spectrum, few studies have examined the relationship between the Self and episodic memory. Those that have focused on the narrative facet of the Self, and indicated a lack of narrative SRE in chronic schizophrenia (56–59). To date, no study has focussed on the minimal facet of the Self in the study of the SRE in the schizophrenia spectrum, including in the FEP.

The primary objective of the present pilot study was to investigate the modulation of the episodic memory of naturalistic events by the minimal Self in FEP, using an immersive virtual reality paradigm. A two-by-two mixed design was developed, comprising two groups of participants (10 FEP patients and 35 matched healthy controls, CTL), with each participant undergoing two experimental conditions in a counterbalanced order (synchronous/asynchronous avatar). In a recognition task, we comprehensively assessed the episodic memory of naturalistic daily life events encoded incidentally in a virtual city while being strongly or weakly embodied in a virtual avatar using the full-body illusion with visuomotor stimulation. Retrieval phenomenology was also assessed. Given that FEP patients experience self-disorders, our hypothesis was that their modulation of the episodic memory by the minimal Self would be altered, resulting in no improvement in episodic memory with a strong embodiment (synchronous avatar) compared to a weak embodiment (asynchronous avatar), in contrast to healthy participants. The secondary objective of the study was to explore the relationships between the sense of Self, sense of embodiment, episodic memory, schizotypal personality traits in healthy participants, and psychopathology in FEP patients.

## 2 Materials and methods

### 2.1 Participants

The present experimental study included 10 FEP patients (6 women/4 men; mean age 19.8 ± 3.4 years) diagnosed within the year based on the criteria of the Comprehensive Assessment of At-Risk Mental State (CAARMS) (60), and 35 gender- and age-matched CTL (21 women/14 men; mean age 22.1 ± 4.2 years), defined by no self-reported neurological or psychiatric history and no current use of medication affecting cognitive function.

The inclusion criteria for both groups were as follows: participants were required to be aged between 16 and 35, to be French-speaking, to have a normal or corrected-to-normal vision, to have no sensorimotor deficits, and to have a low susceptibility to motion sickness as assessed by the Motion Sickness Susceptibility Questionnaire – Short Form (MSSQ) (61). The 16-35 age range was selected for investigation because psychosis typically emerges during late adolescence and early adulthood, which are critical neurodevelopmental stages (26, 62). This makes this age range a crucial window for early detection and intervention. The non-inclusion criteria for both groups were defined in accordance with the recommendations of the ANSES (French Agency for Food, Environmental and Occupational Health and Safety) on the health effects related to exposure to virtual reality and augmented reality technologies (63). These included epilepsy, pregnancy, balance or posture disorders, frequent migraines, and highly anxious temperament as defined by a score above 56 on the Strait-Trait Anxiety Inventory Part B (STAI-B) (64). The following non-inclusion criteria were applied to FEP only: severe and unstable medical conditions; absence of medical insurance; participation in another intervention trial; enforced hospitalisation; intellectual deficiency; former psychotic episode, chronic schizophrenia, schizoaffective, or bipolar disorder; current depression as defined by a total score greater than 34 on the Montgomery-Åsberg Depression Scale (MADRS) (65); having been receiving antipsychotic treatment for more than 12 months; current medication with benzodiazepine exceeding 30 mg per day equivalent diazepam; current moderate to severe substance use disorder (SUD, DSM-IV criteria) except for nicotine, or former severe SUD for more than 5 years.

Patients were recruited among people seeking care in one of the specialised adolescents and young adults consultations at the GHU Paris Psychiatry and Neuroscience, as part of a pilot study for the research protocol SCOPe (‘Self-administered COgnitive Personalised training in early psychosis: A randomised controlled trial in adolescents and young adults to assess efficacy and efficiency of an eHealth application providing personalised cognitive training’). Psychopathological and neuropsychological assessments were conducted by trained clinicians. Healthy controls were recruited among the students at the Institute of Psychology of Université Paris Cité, as well as people who had expressed an interest in participating in cognitive science experiments via the mailing list of the *Relais d’Information sur les Sciences de la Cognition* (RISC).

All participants or their legal representatives provided free and informed written consent. The studies were conducted in accordance with the French regulatory framework and were approved by the relevant ethics committees (for CTL: Comité d’Éthique de la Recherche de l’Université Paris Cité – IRB number: 0012021-107; for FEP: Comité de Protection des Personnes Sud-Méditerranée II – IRB number: 2019-A00399-48).

### 2.2 Equipment

Immersion in the virtual environment was achieved using the HTC Vive Pro virtual reality equipment (Taoyuan City, Taiwan: HTC Corporation), which consisted of a head-mounted display with headphones, two controllers held in each hand to track the positions and movements of the hands, and three trackers worn on the back around the waist and around each ankle to track the positions and movements associated with the pelvis, and the legs and feet.

All self-reported questionnaires and the recognition task were implemented using the Python module *Neuropsydia* (66) and completed by the participants on a laptop.

### 2.3 Virtual environment

**Figure 1:**
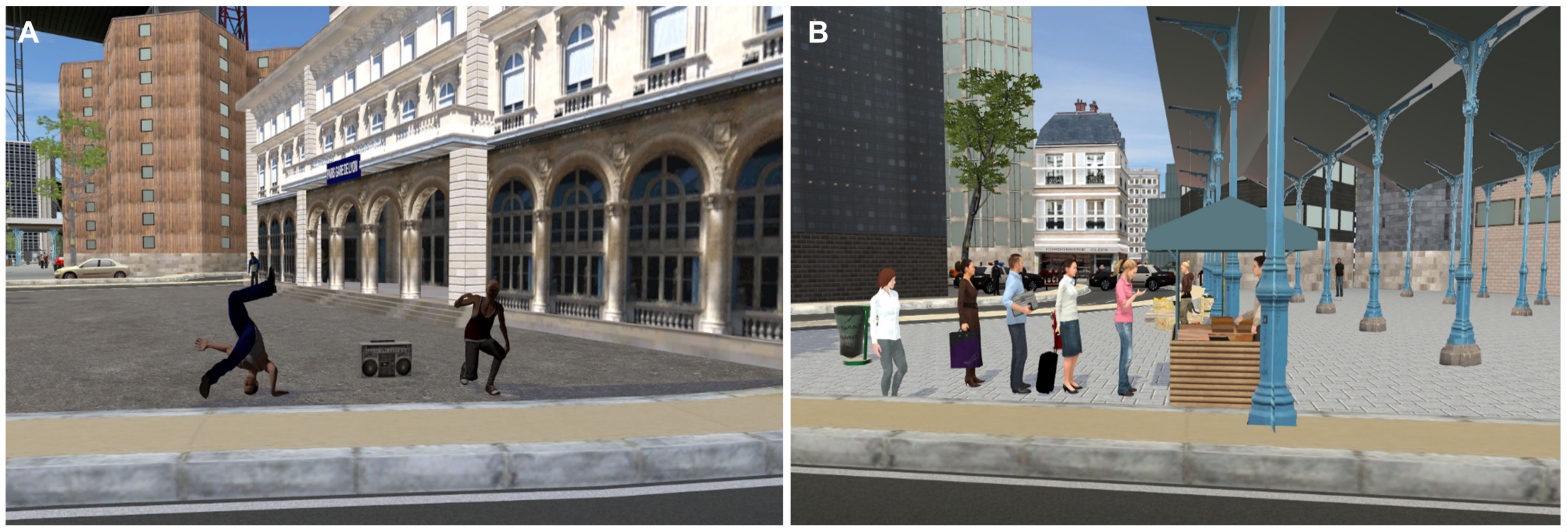
Examples of naturalistic daily life events encountered in the virtual city. **(A)** Two men perform hip-hop dance to rap music in front of a train station. **(B)** A woman gets impatient in a queue at the market and overtakes the other customers.

The virtual environment was created in our laboratory using Unity 2019.2.5f1 software. It consisted of a reproduction of a city, with naturalistic daily life events unfolding along the route. These events featured characters, animals, or objects in specific situations. They were distinguishable from other elements of the environment by the fact that they presented both specific visual and auditory animations. For instance, two men danced hip-hop to rap music outside Paris’s Gare de Lyon station, and an impatient woman overtook other shoppers in a market queue (**Figure 1**). All twenty events are described and illustrated in **Supplementary Table 1**.

### 2.4 Experimental procedure

**Figure 2:**
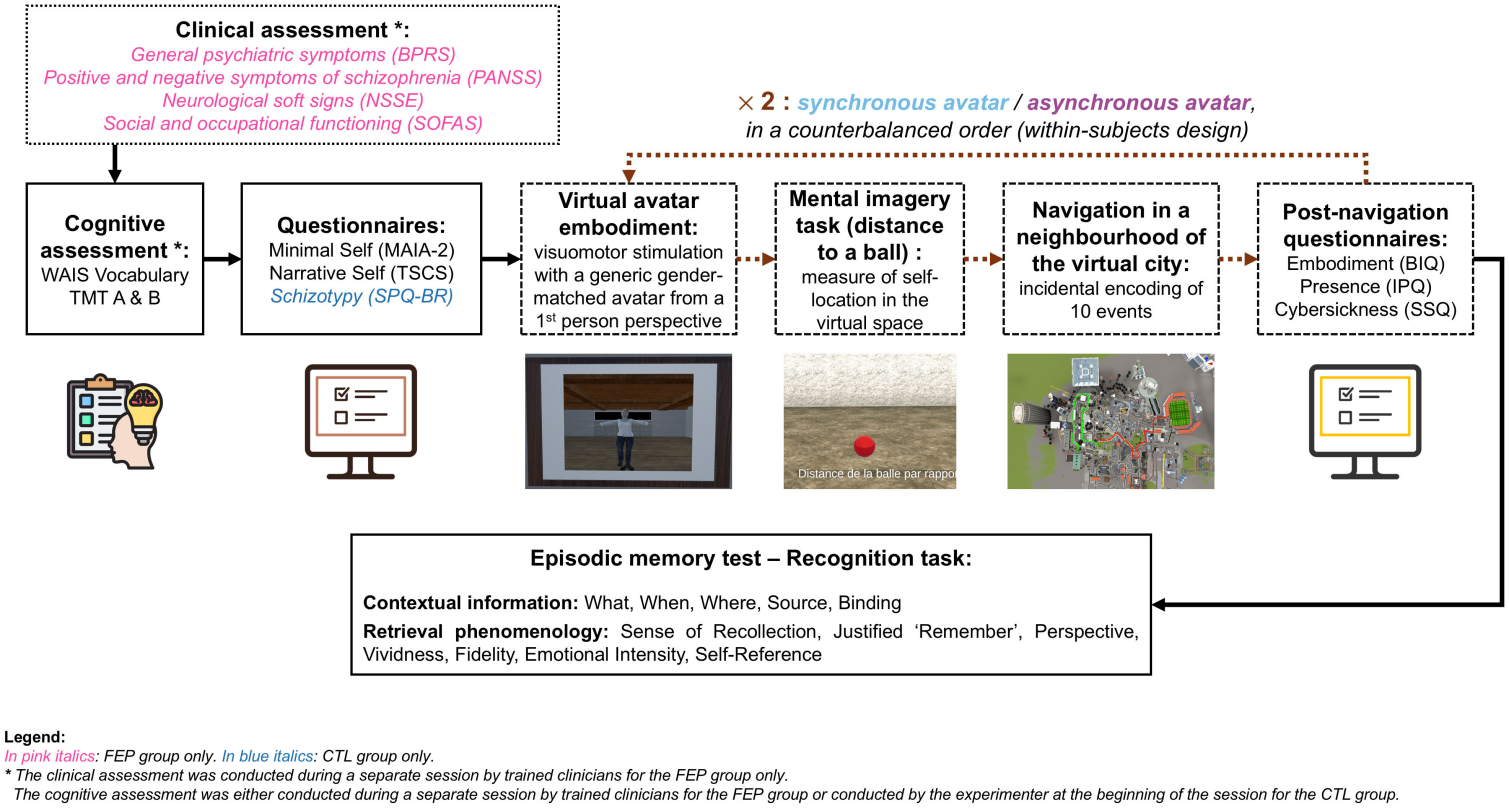
Experimental procedure. Participants first underwent a brief cognitive assessment and completed questionnaires assessing their self-reported sense of Self. They were then given the instructions to read, equipped with the virtual reality equipment, and immersed in the training path of the virtual city. Then, they completed a first embodiment procedure with a generic gender-matched avatar from a first-person perspective, using either synchronous or asynchronous visuomotor stimulation to induce the full-body illusion. Following avatar embodiment induction, they completed a mental imagery task measuring their self-location in the virtual space, and navigated in a first neighbourhood of the virtual city where they encoded ten daily life events incidentally. After the navigation, they completed three questionnaires assessing their sense of embodiment, sense of presence, and cybersickness. Then, the avatar embodiment induction, mental imagery task, navigation in another neighbourhood of the virtual city, and post-navigation questionnaires, were repeated a second time with the alternate avatar synchrony condition, in a counterbalanced order. Finally, participants completed an episodic memory recognition task assessing the contextual information of the events and retrieval phenomenology.

The experimental procedure, which is summarised in **Figure 2**, was adapted from a previous study conducted in our laboratory (45).

#### 2.4.1 Experimental conditions

The study employed a two-by-two mixed design, with two groups of participants (FEP/CTL), and each participant undergoing two experimental conditions (synchronous avatar/asynchronous avatar). The order of the avatar synchrony conditions was counterbalanced across participants.

#### 2.4.2 Neuropsychological assessment

At the beginning of the session for the CTL, or in a separate session for the FEP, participants were administered two neuropsychological tests by the experimenter for the CTL or by trained clinicians for the FEP. These were the Vocabulary subtest from the Wechsler Adult Intelligence Scale – 4^th^ edition (WAIS-IV) (67), which assessed verbal comprehension abilities, and the Trail Making Test Parts A and B (68), which assessed executive functioning as cognitive flexibility (TMT B-A). The scores were standardised in accordance with the norms established in previous studies (67, 69).

#### 2.4.3 Sense of self assessment

Following the neuropsychological assessment for the CTL, or at the beginning of the session for the FEP, participants completed self-reported questionnaires to assess their sense of minimal Self and sense of narrative Self. The Multidimensional Assessment of Interoceptive Awareness – Version 2 (MAIA-2) (70, 71), and the Tennessee Self-Concept Scale – Second Edition, Short Form (TSCS-2) (72, 73) were employed, respectively. The MAIA comprised 37 items rated on 6-point Likert scales ranging from 0 (‘Never’) to 5 (‘Always’), with some reversed items, assessing eight dimensions, namely: Noticing (4 items), reflecting the awareness of uncomfortable, comfortable, and neutral body sensations; Not-Distracting (6 items), reflecting the tendency not to ignore or distract oneself from sensations of pain or discomfort; Not-Worrying (5 items), reflecting the tendency not to worry or experience emotional distress with sensations of pain or discomfort; Attention Regulation (7 items), reflecting the ability to sustain and control attention to body sensations; Emotional Awareness (5 items), reflecting the awareness of the connection between body sensations and emotional states; Self-Regulation (4 items), reflecting the ability to regulate distress by attention to body sensations; Body Listening (3 items), reflecting active listening to the body for insight; and Trust (3 items), reflecting the experience of one’s body as safe and trustworthy. In its short form, the TSCS-2 comprised 21 items rated on 5-point Likert scales ranging from 1 (‘No, not at all’) to 5 (‘Yes, completely’), with some reversed items, providing: two global scores, namely a Total score reflecting global self-esteem, and a Conflict score reflecting ambivalence; two validity scores, namely Inconsistency reflecting the internal consistency among responses to similar items, and Response Distribution reflecting the flexibility of the self-concept; and five scores representing several facets of the narrative Self, which are the Physical (5 items), Moral (4 items), Personal (5 items), Familial (4 items), and Social (3 items) representations of the Self.

#### 2.4.4 Psychopathological assessment and medication

Immediately after the questionnaires assessing the sense of Self, CTL completed the Schizotypal Personality Questionnaire – Brief Revised (SPQ-BR) (74, 75), which assessed schizotypal personality traits. It comprised 32 items rated on dichotomous scales coded as 1 (‘Yes’) and 0 (‘No’), assessing Positive (14 items), Negative (10 items), and Disorganisation (8 items) dimensions. Patients’ psychopathological assessment was conducted by trained clinicians in a separate session: the Brief Psychiatric Rating Scale – Expanded (BPRS-E) (76, 77) assessed general psychiatric symptoms; the Positive And Negative Symptoms Scale (PANSS) (78, 79) assessed positive and negative symptoms of schizophrenia; the Neurological Soft Signs Examination (NSSE) (80) assessed subtle integrative neurological anomalies; and the Social and Occupational Functioning Assessment Scale (SOFAS) (81) assessed routine social functioning. Except for the SOFAS, higher scores indicated greater impairment. Patients’ current antipsychotic medication was converted to chlorpromazine-equivalent doses using the R *chlorpromazineR* package (82), based on the conversion factors from Leucht et al. (83).

#### 2.4.5 Instructions and scenario

**Figure 3:**
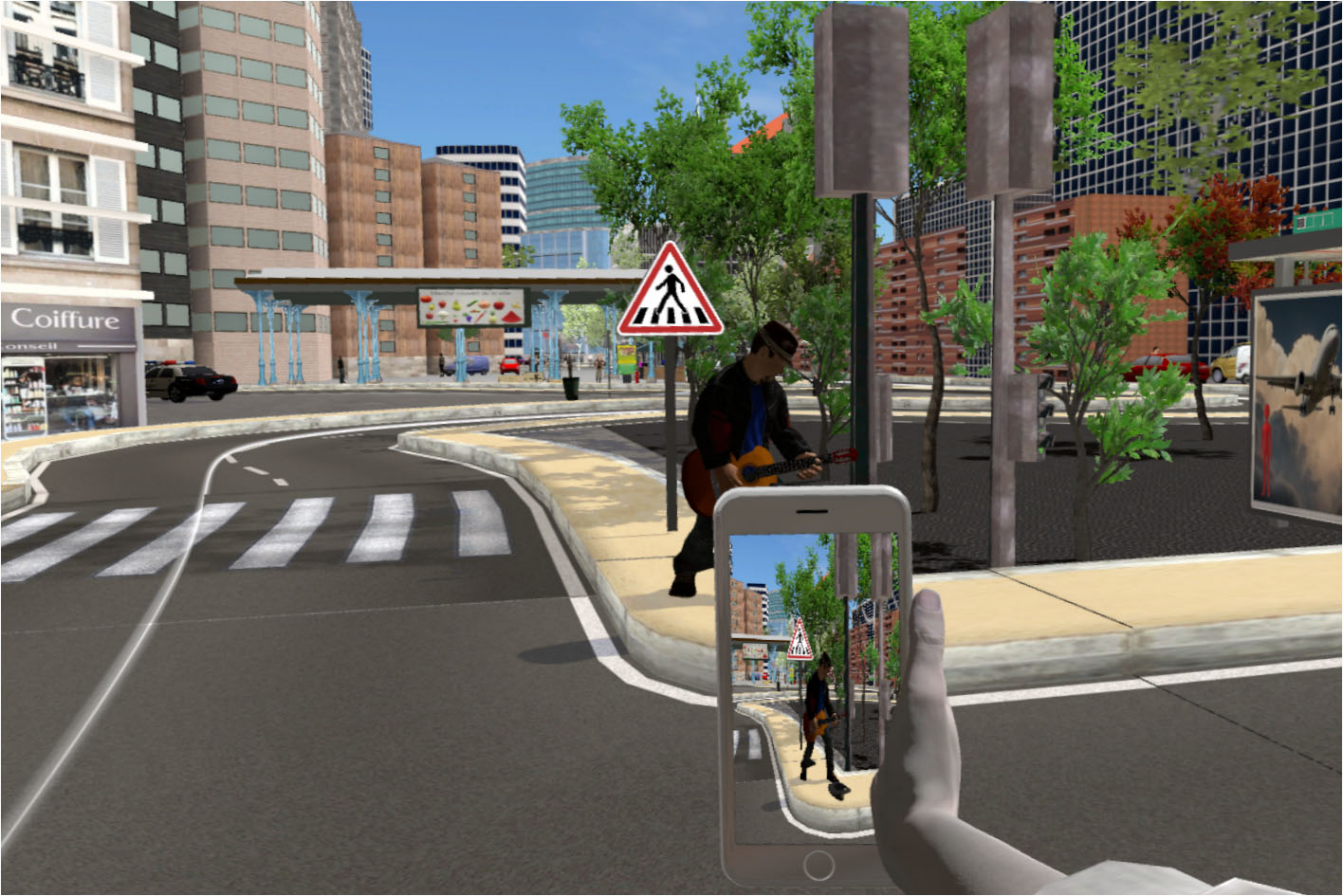
Example of a photography using the smartphone to reinforce the visuomotor stimulation throughout the navigations.

Prior to their immersion in the virtual environment, participants were provided with a sheet containing the instructions for the experiment. They were informed that they would be immersed successively in two virtual environments, which corresponded to different neighbourhoods of a city. They were instructed to walk around these two environments, following a path indicated by a white line on the ground, as if they were visiting different neighbourhoods to move in there. Furthermore, they were instructed to explore each environment at their own pace and to be mindful of their surroundings. Additionally, participants were encouraged to take photographs of anything they deemed noteworthy within the environment, using the smartphone held in the avatar’s right hand (**Figure 3**). They were also warned that they would frequently encounter mirrors or reflective shop windows within each neighbourhood, and that they should pause in front of them and move their arms and legs alternately, observing their body in the mirror and directly, before continuing their way. Finally, participants were provided with instructions on how to navigate in the virtual environment. This involved touching the directional pad located on the right controller and orienting their head in the direction of the intended destination.

#### 2.4.6 Training

After reading the instructions, the participants were positioned in the centre of the room and equipped with virtual reality equipment. They were first immersed in the training path of the virtual environment with a non-visible avatar, allowing them to become acquainted with the virtual reality system, the navigation modalities, the virtual city, and the instructions for the experiment. The training path comprised three naturalistic daily life events that were distinct from the events occurring in the paths used for incidental memory encoding.

#### 2.4.7 Induction of avatar embodiment using a visuomotor stimulation

**Figure 4:**
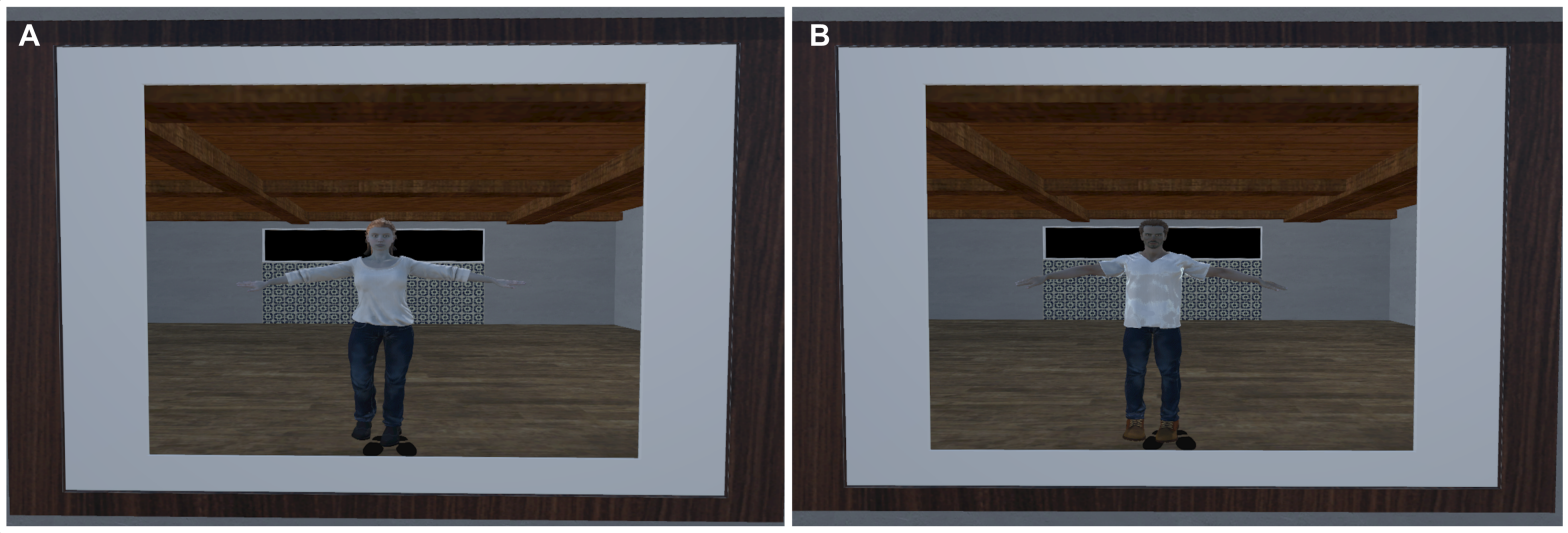
Female **(A)** and male **(B)** generic avatars in front of the virtual mirror for the induction of the full-body ownership illusion.

Prior to each of the two navigations in the virtual city, a full-body ownership illusion was induced in a generic gender-matched avatar seen from a first-person perspective, using a visuomotor stimulation in front of a virtual mirror (45, 50, 51, 54) (**Figure 4** and **Supplementary Video S1**). For one minute, participants were instructed to alternately move their heads, right arms, left arms, left legs, and right legs, making wide movements and observing their bodies both in the mirror and directly, alternately. In one condition, the visuomotor stimulation was synchronous, aiming to induce a strong sense of embodiment over the avatar. In the alternative condition, the visuomotor stimulation was asynchronous, aiming to induce a weak sense of embodiment over the avatar. A temporal delay of 650 ms was introduced between the seen movements of the avatar in the virtual environment and the movements performed by the participants in real life. The choice of the delay was based on a previous study (45).

Following this embodiment induction, the virtual environment was instantly switched to a new, uncluttered environment, only containing a red ball. This environment enabled participants to complete a mental imagery task adapted from Nakul et al. (84), designed to measure their perceived self-location in the virtual space, thus assessing their perceived space occupation by their bodies. In this task, a virtual red ball was positioned in front of the avatar, at a distance of nine metres. The ball then started rolling following a linear trajectory towards the participants, at a constant velocity of 1 m.s^-1^ for a duration of six seconds. During the trajectory, the screen would become black, and participants were instructed to imagine that the ball was continuing to roll towards them at the same speed, and to indicate when they believed the ball had reached their feet by pressing the trigger on the back of the right-hand controller. The distance to the ball was recorded as a measure of the participants’ perceived occupation of the virtual space by their bodies, corresponding to the distance, in metres, between the position of the avatar and the point where the participants stopped the ball. The distance to the ball was estimated as the average distance recorded over three trials.

#### 2.4.8 Navigation in the virtual city and incidental encoding of naturalistic daily life events

Following the avatar embodiment induction and mental imagery task with a synchronous or asynchronous avatar, participants were immersed with their synchronous or asynchronous avatar, respectively, in one of the two neighbourhoods of the virtual city. Each neighbourhood comprised ten naturalistic daily life events, which constituted the stimuli that aimed to be encoded incidentally in episodic memory. Therefore, participants encoded ten events while being embodied in a synchronous avatar, and ten other events while being in an asynchronous avatar, for a total of twenty encoded events. The order of the two neighbourhoods was counterbalanced across participants to reduce potential systematic bias associated with specific event characteristics. Furthermore, reflective surfaces were encountered every three or four events in each neighbourhood, aiming to reinforce the synchronous or asynchronous visuomotor stimulation throughout the navigations. The possibility of taking photographs during the navigations was also designed to reinforce the visuomotor stimulation. Indeed, participants had to raise their right arm to view the smartphone screen and take photographs, thereby inducing a synchronous or asynchronous visuomotor stimulation each time the arm was raised. Additionally, participants were able to hear a camera shutter sound each time they pressed the trigger to take a photograph. In the synchronous avatar condition, the sound was heard at the moment they pressed the trigger; in the asynchronous avatar condition, the sound was heard with a 650 ms delay corresponding to the visuomotor asynchrony delay. The duration of navigation was recorded in each part of the city. An excerpt from a navigation in the virtual city has been provided in the **Supplementary Material** (**Supplementary Video S2**).

#### 2.4.9 Post-navigation measures: embodiment, presence, and cybersickness

Following each navigation in the virtual environment, participants completed three self-reported questionnaires. The Body-Illusion Questionnaire (BIQ) (45) was used to assess the sense of embodiment. It comprised three items assessing agency, body ownership, and self-location, respectively, as well as two control items assessing compliance and suggestibility. The statements were rated on 7-point Likert scales, ranging from –3 (‘Strongly disagree’) to +3 (‘Strongly agree’). The IGroup Presence Questionnaire (IPQ) (85) was used to assess the sense of presence. It comprised fourteen items rated on 7-point Likert scales ranging from 0 (‘Not at all’) to 6 (‘Completely’), with some reversed items, assessing four dimensions of presence (General presence: 1 item, Spatial presence: 5 items, Involvement: 4 items, Realness: 4 items). The Simulator Sickness Questionnaire (SSQ) (86, 87) was used to assess cybersickness. It comprised sixteen items rated on 4-point Likert scales ranging from 0 (‘Not at all’) to 3 (‘Severely’), assessing two dimensions of cybersickness (Nausea: 9 items, Oculomotor: 7 items).

#### 2.4.10 Virtual reality episodic memory test: comprehensive recognition task

Finally, the episodic memory of the naturalistic daily life events encountered was assessed with a comprehensive recognition task. Participants were presented with a series of pictures of the twenty daily life events encountered in the virtual city, interspersed with ten additional lures, in a randomised order. The background of each picture was rendered white to eliminate any contextual cues. Participants were first asked to indicate whether they had encountered each event (Yes/No judgement). Then, for each correctly recognised event, participants were asked to rate a first item assessing retrieval phenomenology on a scale from 0 to 100, namely their sense of recollection, based on the Remember/Know paradigm (32). The instructions were designed to assess subjective re-experiencing as a proxy for autonoetic consciousness, and are provided in **Table 1**. The endpoints of the scale were defined as ‘100 – I remember’ and ‘0 – I know’, based on previous studies that used Likert scales rather than a dichotomous choice between ‘Remember’ and ‘Know’ (88, 89).

For each correctly recognised event associated with a strong sense of recollection (i.e., a score above 50 on the Remember/Know scale), contextual information was then assessed. Participants were asked to indicate the source of the memory (by selecting one of two options: first navigation/second navigation) and to provide objective spatiotemporal information about the event, i.e. where the event was located in relation to the participant (three options: to my left/to my right/in front of me) and when it occurred (three pictures of events were displayed, including the event being assessed; participants were asked to place these events in the correct temporal sequence in which they occurred in the city). Finally, participants were asked to rate on scales from 0 to 100 five other items assessing retrieval phenomenology, i.e. memory perspective (from 3^rd^ to 1^st^ person), vividness, fidelity, emotional intensity, and self-reference. The specific instructions for these items are provided in **Table 1**.

**Table 1:** English translation of items from the episodic memory test assessing retrieval phenomenology.

Retrieval phenomenology dimension	Item
Sense of recollection	To what extent can you re-experience the specific event with its context? ‘I remember’: this characterises your memory if you can recall the event within its specific encoding context and re-experience the specific event. ‘I know’: this characterises your memory if you know that you have encountered the event but are not able to mentally re-experience the scene.
Perspective	From what point of view do you remember this event? In 1^st^ person: you are observing the scene as an actor in the scene, from the point of view of your body. In 3^rd^ person: you are observing the scene from the point of view of an outside observer, your body is an integral part of the scene.
Vividness	How clear is the mental image you have of this event?
Fidelity	How accurate do you think your mental representation of the event is?
Emotional intensity	How intense do you feel the emotions (positive or negative) associated with the memory of this event?
Self-reference	To what extent does this event refer to important aspects of your personality?

The recognition task was scored as follows. For each correctly recognised event associated with a strong sense of recollection, a score of binding was calculated: the binding score was equal to 3 when participants correctly recognised the event and gave a correct answer to the questions ‘Where’ and ‘When’; the binding was equal to 2 when participants correctly recognised the event and gave a correct answer either to the question ‘Where’ or to the question ‘When’; the binding was equal to 1 when participants correctly recognised the event but failed to answer both questions ‘Where’ and ‘When’. Global scores were then computed for each condition. *Accuracy* corresponded to the number of hits (i.e., correctly recognised events) divided by the number of events in each condition (i.e., 10 events), expressed as a percentage. *Sensitivity* estimated the ability to discriminate between events encountered in the virtual environment and lures that were not encountered, using the non-parametric index of sensitivity ‘A prime’ (90), computed with the *dprime* function from the R package *psycho* (91). *Source* corresponded to the number of correct answers to the question ‘Source,’ divided by the number of events correctly recognised in each condition, expressed as a percentage. *Where* corresponded to the number of correct answers to the question ‘Where’, divided by the number of events correctly recognised in each condition, expressed as a percentage. *When* corresponded to the number of correct answers to the question ‘When’, divided by the number of events correctly recognised in each condition, expressed as a percentage. *Mean Binding* was calculated as the mean of the binding scores. *WWW Binding* (‘What-When-Where’ Binding) was another approach to calculating the global binding for each condition, rather than for each event. This was achieved by summing the number of correct ‘What’, ‘When’, and ‘Where’ answers for each condition, and dividing this sum by the maximum possible value of this sum (i.e., 30), expressed as a percentage, enabling the binding score to be expressed as a function of the number of events correctly recognised. *Sense of Recollection* corresponded to the mean score on the Remember/Know scale, ranging from 100 to 0. *Justified ‘Remember’* corresponded to the proportion of events associated with a recollection score above 50 and a binding of 3, indicating a sense of remembering justified by correct contextual information. Finally, a mean score was calculated for each retrieval phenomenology score, ranging from 0 to 100.

### 2.5 Statistical analyses

Statistical analyses were conducted using *R* version 4.3.3 (92). All significance tests were based on α = 0.05. Descriptive statistics were computed using the *compareGroups* package (93). Robust methods were used due to the small sample size for FEP. Between-group comparisons of sociodemographic data, neuropsychological test scores, and sense of Self scores were computed using robust methods for comparing independent means, with the Yuen test based on trimmed means and bootstrapping, using the *WRS2* package (94). To investigate main effects of *Group* (FEP/CTL) and *Avatar synchrony* (synchronous/asynchronous), and their interaction effect, two-way mixed analysis of variance (ANOVA) were computed using robust methods based on trimmed means and bootstrapping (95), using the *WRS2* package (94). Follow-up analyses for significant interactions were conducted using the *stats* package (92). Wilcoxon signed-rank tests were used to compare the synchronous and asynchronous avatar conditions within the CTL and FEP groups separately. Mann-Whitney tests were used to compare the CTL and FEP groups within each avatar condition separately. Effect sizes were reported as rank-biserial correlations (*r_rb_*). Spearman’s correlations were computed separately for the FEP and CTL groups, using the *stats* (92) and *psych* (96) packages, and adjusted for multiple comparisons using the Benjamini-Hochberg method (97). The graphs were plotted using the packages *ggplot2* (98), *ggpubr* (99), and *corrplot* (100).

## 3 Results

### 3.1 Sample description

Regarding sociodemographic data, FEP and CTL were matched for age and sex. However, CTL presented significantly more years of education than FEP. Regarding neuropsychological data, no significant group difference was observed in verbal comprehension (WAIS Vocabulary) nor executive functioning (TMT B-A). Regarding the self-reported sense of Self, there was no significant difference between FEP and CTL for the sense of minimal Self (MAIA Total and subscores). However, FEP presented significantly weaker self-esteem than CTL (TSCS Total), a more petrified narrative Self (TSCS Response distribution), and a poorer moral representation of their Self (TSCS Moral) (**Table 2**). Regarding psychopathology, for FEP, BPRS Total score indicated a mild severity of psychiatric symptoms; PANSS Total score indicated a mild severity of schizophrenia symptoms globally, with a mild severity of positive symptoms and of general psychopathology, and a moderate severity of negative symptoms; NSSE Total score indicated moderate severity of subtle neurological abnormalities, with minimal impairments in motor integration, sensory integration, involuntary movements, and lateralisation, and mild impairment in motor coordination; SOFAS scores indicated moderate difficulties in social, professional, or academic functioning over the past year and month. For CTL, SPQ Total score indicated moderate levels of schizotypy, with low levels of positive schizotypy, and moderate levels of negative schizotypy and disorganisation symptoms.

**Table 2:** Summary statistics for sociodemographic data, neuropsychological test scores, sense of Self questionnaires, and psychopathological data, by Group.

	FEP N = 10	CTL N = 35	*Yuen test*		
	*Mean (SD)*	*Mean (SD)*	*M_diff_*	*Y_t_*	*p*
Sociodemographic					
Age (years)	19.8 (3.39)	22.1 (4.24)	1.76 [-1.35, 4.87]	1.32	0.24
Sex:					
Female	6 (60.0%)	21 (60.0%)			
Male	4 (40.0%)	14 (40.0%)			
Years of education	11.9 (2.13)	14.1 (2.12)	2.10 [0.91, 3.28]	3.69	**0.003**
Neuropsychology					
WAIS Vocabulary (age-scaled score) (/19)	10.3 (3.47)	9.57 (3.54)	-0.52 [-5.39, 4.34]	-0.33	0.75
TMT B-A (Z-score)	1.15 (1.27)	1.39 (3.62)	-0.91 [-2.31, 0.49]	-1.57	0.18
Sense of Self					
MAIA-2 – Total (/40)	21.6 (5.03)	23.8 (3.28)	0.99 [-2.30, 4.29]	0.77	0.48
MAIA-2 – Noticing (/5)	3.12 (0.68)	3.53 (0.73)	0.31 [-0.25, 0.87]	1.24	0.23
MAIA-2 – Not-Distracting (/5)	2.38 (0.65)	2.06 (0.72)	-0.30 [-0.90, 0.31]	-1.20	0.27
MAIA-2 – Not-Worrying (/5)	2.34 (1.03)	2.59 (0.99)	0.44 [-0.27, 1.15]	1.44	0.19
MAIA-2 – Attention Regulation (/5)	2.49 (0.81)	2.90 (0.67)	0.31 [-0.19, 0.81]	1.29	0.22
MAIA-2 – Emotional Awareness (/5)	3.10 (1.00)	3.59 (0.87)	0.5 [-0.13, 1.14]	1.77	0.11
MAIA-2 – Self-Regulation (/5)	2.58 (1.06)	2.88 (0.71)	0.36 [-0.83, 1.56]	0.93	0.40
MAIA-2 – Body Listening (/5)	2.07 (0.90)	2.66 (1.00)	0.49 [-0.18, 1.17]	1.68	0.13
MAIA-2 – Trust (/5)	3.50 (1.52)	3.62 (1.11)	-0.04 [-1.76, 1.69]	-0.06	0.96
TSCS-2 –Total (/105)	76.3 (9.70)	85.3 (8.31)	10.14 [3.05, 17.24]	3.34	**0.01**
TSCS-2 – Conflict (/-15)	-13.90 (4.84)	-12.29 (5.02)	1.17 [-2.88, 5.21]	0.69	0.52
TSCS-2 – Inconsistency (/4)	1.10 (0.88)	0.86 (0.97)	-0.38 [-0.84, 0.08]	-1.67	0.10
TSCS-2 – Response Distribution (/21)	5.80 (3.94)	9.49 (3.95)	4.52 [-0.16, 9.21]	2.75	**0.05**
TSCS-2 – Physical (/25)	17.5 (2.68)	19.1 (2.83)	1.64 [-0.62, 3.9]	1.86	0.11
TSCS-2 – Moral (/20)	13.6 (1.96)	16.4 (1.77)	3.14 [1.13, 5.16]	3.98	**0.01**
TSCS-2 – Personal (/25)	17.2 (3.85)	19.9 (2.72)	2.98 [-0.28, 6.23]	2.13	0.07
TSCS-2 – Familial (/20)	17.3 (2.54)	18.0 (2.66)	1.05 [-1.28, 3.38]	1.09	0.33
TSCS-2 – Social (/15)	10.7 (2.45)	11.8 (2.02)	1.14 [-1.75, 4.04]	1.12	0.33
Psychopathology					
BPRS – Total (/168)	51.3 (11.4)				
PANSS – Total (/210)	72.9 (16.0)				
PANSS – Positive (/49)	14.5 (5.44)				
PANSS – Negative (/49)	21.7 (6.60)				
PANSS – General psychopathology (/112)	36.7 (7.44)				
NSSE – Total (/69)	12.3 (6.05)				
NSSE – Motor coordination (/24)	6.67 (4.34)				
NSSE – Motor integration (/18)	1.39 (1.24)				
NSSE – Sensory integration (/15)	2.19 (1.87)				
NSSE – Involuntary movements (/6)	0.39 (0.70)				
NSSE – Lateralisation (/6)	0.56 (0.73)				
SOFAS – Last year (/100)	50.6 (7.65)				
SOFAS – Last month (/100)	53.7 (8.83)				
SPQ – Total (/32)		11.7 (6.08)			
SPQ – Positive (/14)		3.65 (2.90)			
SPQ – Negative (/10)		4.29 (2.81)			
SPQ – Disorganisation (/8)		3.79 (2.11)			
Medication					
Chlorpromazine equivalent dose (mg)	616 (574)				

FEP, first-episode psychosis patients; CTL, healthy controls; M_diff_, trimmed mean difference with 95% confidence interval; Y_t_, Yuen test statistic; p, Yuen test p-value; WAIS, Wechsler Adult Intelligence Scale; TMT, Trail Making Test; MAIA-2, Multidimensional Assessment of Interoceptive Awareness – Version 2; TSCS-2, Tennessee Self-Concept Scale – Second Edition, Short Form. BPRS, Brief Psychiatric Rating Scale – Expanded; PANSS, Positive And Negative Symptoms Scale; NSSE, Neurological Soft Signs Examination; SOFAS, Social and Occupational Functioning Assessment Scale; SPQ, Schizotypal Personality Questionnaire – Brief Revised. In bold: statistically significant results.

### 3.2 Sense of presence, cybersickness, and navigation duration

The results of the robust ANOVA indicated that there was no significant interaction effect and no significant main effect of *Group* on the sense of presence (IPQ Total). However, there was a significant main effect of *Avatar synchrony*, indicating that the synchronous avatar was associated with a higher sense of presence than the asynchronous avatar, independently of the group. Additionally, IPQ Realness was also significantly higher with the synchronous avatar than with the asynchronous avatar. Regarding cybersickness, no significant interaction effect or main effects were observed on the SSQ Total score or subscores, indicating that there were no differences in cybersickness across groups and conditions. Finally, a significant interaction effect and a significant main effect of *Avatar synchrony* were observed on navigation duration. Follow-up analyses indicated that the asynchronous avatar was associated with a significantly longer navigation than the synchronous avatar for CTL (*W* = 443.5, *p* = .013, *r_rb_* = 0.49). There was no significant difference between avatar synchrony conditions for FEP, and no significant difference between groups across conditions (**Table 3**).

**Table 3:** Sense of presence, cybersickness, and navigation duration, by Group and Avatar synchrony.

	FEP		CTL		Robust ANOVA		
	Async.	Sync.	Async.	Sync.	Main effect of *Group*	Main effect of *Avatar synchrony*	Interaction effect
	*Mean (SD)*	*Mean (SD)*	*Mean (SD)*	*Mean (SD)*	*Q_a_, p*	*Q_b_, p*	*Q_ab_, p*
Sense of presence							
IPQ – Total (/84)	43.4 (10.1)	47.2 (9.33)	42.3 (13.5)	47.0 (12.6)	*Q_a_* = -1.97 *p* = .63	***Q_b_* = -3.69** ***p* = .01**	*Q_ab_* = 0.14 *p* = .96
IPQ – General Presence (/6)	3.40 (0.97)	3.60 (0.97)	3.34 (1.39)	3.86 (1.44)	*Q_a_* = 0.10 *p* = .84	*Q_b_* = -0.34 *p* = .46	*Q_ab_* = -0.38 *p* = .23
IPQ – Spatial Presence (/30)	18.4 (3.06)	18.8 (3.97)	18.8 (4.98)	19.9 (4.57)	*Q_a_* = 0.43 *p* = .76	*Q_b_* = -1.22 *p* = .06	*Q_ab_* = 0.003 *p* = .99
IPQ – Involvement (/24)	12.1 (5.26)	13.7 (3.89)	11.7 (4.42)	12.8 (4.33)	*Q_a_* = -1.48 *p* = .39	*Q_b_* = -0.81 *p* = .33	*Q_ab_* = 1.33 *p* = .44
IPQ – Realness (/24)	9.50 (3.84)	11.1 (4.53)	8.46 (6.03)	10.5 (5.31)	*Q_a_* = -0.15 *p* = .94	***Q_b_* = -1.43** ***p* = .01**	*Q_ab_* = -1.34 *p* = .34
Cybersickness							
SSQ – Total (/48)	8.70 (7.33)	8.10 (6.62)	7.71 (7.90)	8.83 (9.26)	*Q_a_* = -2.92 *p* = .43	*Q_b_* = -0.08 *p* = .87	*Q_ab_* = -0.92 *p* = .45
SSQ – Nausea (/27)	3.70 (3.30)	3.40 (3.37)	4.06 (4.96)	4.37 (5.50)	*Q_a_* = -1.68 *p* = .41	*Q_b_* = 0.05 *p* = .84	*Q_ab_* = -0.33 *p* = .56
SSQ – Oculomotor (/21)	5.00 (4.52)	4.70 (3.74)	3.66 (3.45)	4.46 (4.13)	*Q_a_* = -0.96 *p* = .68	*Q_b_* = -0.03 *p* = .94	*Q_ab_* = -0.44 *p* = .65
Navigation duration							
Navigation duration (seconds)	394 (161)	408 (171)	439 (136)	404 (154)	*Q_a_* = 49.86 *p* = .40	***Q_b_* = 40.07** ***p* = .01**	***Q_ab_* = 84.27** ***p* = .05**

FEP, first-episode psychosis; CTL, healthy controls; Async., asynchronous avatar; Sync., synchronous avatar; IPQ, IGroup Presence Questionnaire; SSQ, Simulator Sickness Questionnaire; Q, robust ANOVA statistic; p, robust ANOVA p-value. In bold: statistically significant results.

### 3.3 Sense of embodiment

**Figure 5:**
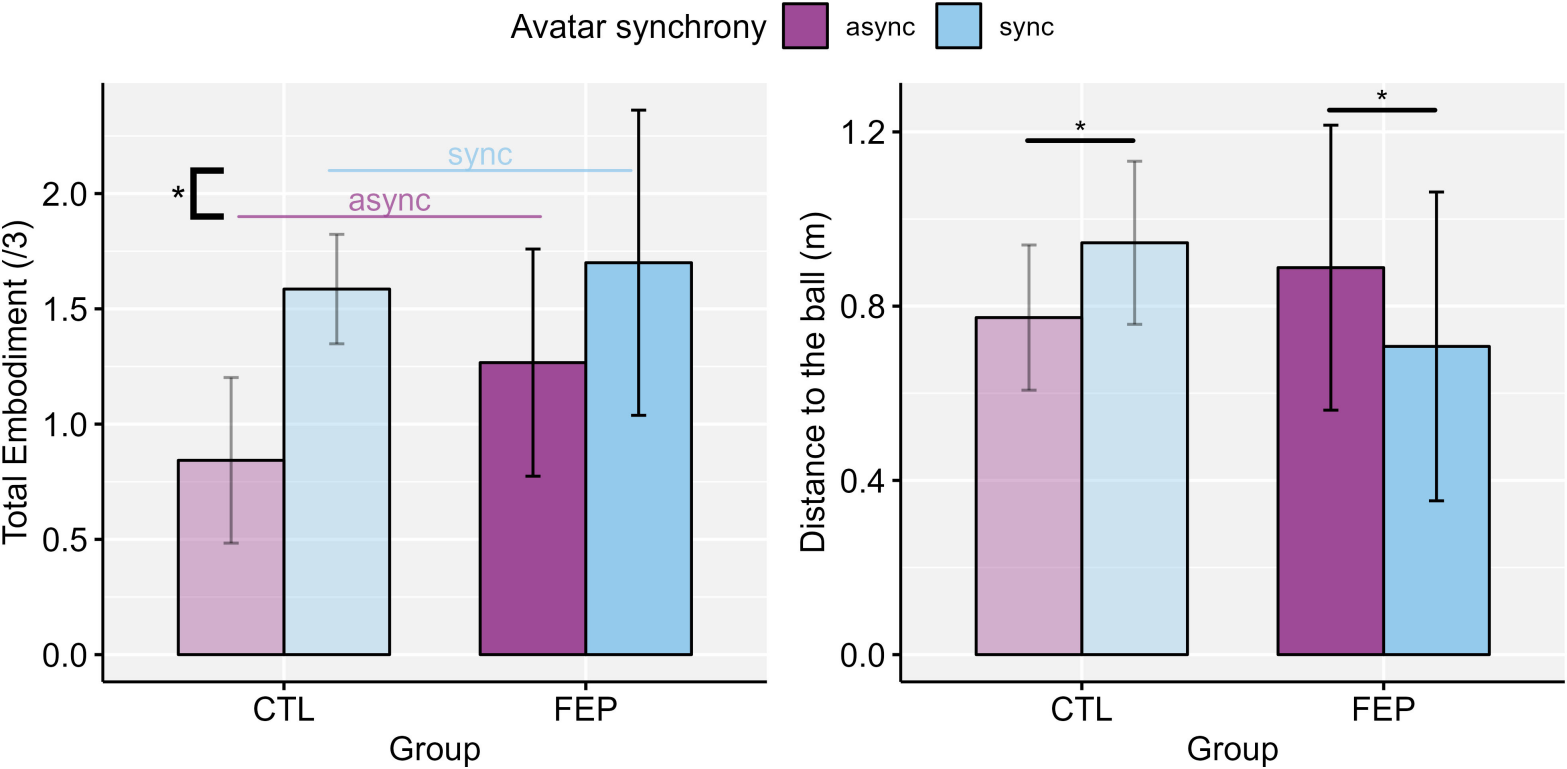
Significant main effect of Avatar synchrony on total sense of embodiment, and significant interaction effect between Group and Avatar synchrony on the distance to the ball in the virtual space. FEP, first-episode psychosis; CTL, healthy controls; Async., asynchronous avatar; Sync., synchronous avatar. Error bars represent the standard error (SE). *: p <.05.

The results of the robust ANOVA indicated that there was no significant interaction effect and no main effect of *Group* on the total embodiment score (BIQ Total) or on the different components of embodiment. However, there was a significant main effect of *Avatar synchrony* on the BIQ Total score, indicating that participants experienced a stronger sense of embodiment with the synchronous avatar than the asynchronous avatar, independently of the group. Additionally, there was a significant interaction between *Group* and *Avatar synchrony* on the distance to the ball. Follow-up analyses indicated that the distance to the ball was significantly greater with the synchronous than the asynchronous avatar for CTL (*W* = 417.0, *p* = .048, *r_rb_* = 0.32). Conversely, the distance to the ball was significantly smaller with the synchronous than the asynchronous avatar for FEP (*W* = 10.0, *p* = .042, *r_rb_* = -0.64). There was no significant difference between groups across conditions (**Table 4** and **Figure 5**).

**Table 4:** Sense of embodiment, by Group and Avatar synchrony.

	FEP		CTL		Robust ANOVA		
	Async.	Sync.	Async.	Sync.	Main effect of *Group*	Main effect of *Avatar synchrony*	Interaction effect
	*Mean (SD)*	*Mean (SD)*	*Mean (SD)*	*Mean (SD)*	*Q_a_, p*	*Q_b_, p*	*Q_ab_, p*
BIQ – Total (/3)	1.27 (1.56)	1.70 (2.09)	0.84 (2.12)	1.59 (1.40)	*Q_a_* = -0.18 *p* = .82	***Q_b_* = -0.47** ***p* = .03**	*Q_ab_* = 0.33 *p* = .74
BIQ – Agency (/3)	1.10 (1.45)	1.40 (1.07)	1.14 (1.77)	1.69 (1.30)	*Q_a_* = 0.51 *p* = .25	*Q_b_* = -0.36 *p* = .14	*Q_ab_* = -0.08 *p* = .86
BIQ – Ownership (/3)	-0.40 (2.01)	0.80 (1.75)	-0.57 (2.02)	0.37 (1.75)	*Q_a_* = -0.20 *p* = .98	*Q_b_* = -0.14 *p* = .78	*Q_ab_* = 0.96 *p* = .47
BIQ – Self-location (/3)	0.70 (1.34)	1.70 (1.34)	0.46 (1.84)	1.03 (1.64)	*Q_a_* = 0.04 *p* = .96	*Q_b_* = -0.24 *p* = .41	*Q_ab_* = 0.88 *p* = .22
BIQ – Control 1 (/3)	-1.20 (1.81)	-0.80 (1.81)	-0.23 (1.88)	-0.06 (2.01)	*Q_a_* = 0.86 *p* = .42	*Q_b_* = -0.19 *p* = .39	*Q_ab_* = 0.25 *p* = .53
BIQ – Control 2 (/3)	-0.40 (1.78)	0.00 (1.63)	-0.77 (2.14)	-1.06 (1.95)	*Q_a_* = -0.94 *p* = .19	*Q_b_* = -0.10 *p* = .70	*Q_ab_* = -0.13 *p* = .80
Distance to the ball (meters)	0.89 (1.03)	0.71 (1.12)	0.77 (0.99)	0.95 (1.11)	*Q_a_* = -0.13 *p* = .78	*Q_b_* = -0.09 *p* = .42	***Q_ab_* = -0.43** ***p* = .02**

FEP, first-episode psychosis; CTL, healthy controls; Async., asynchronous avatar; Sync., synchronous avatar; BIQ, Body-Illusion Questionnaire; Q, robust ANOVA statistic; p, robust ANOVA p-value. In bold: statistically significant results.

### 3.4 Episodic memory

**Figure 6:**
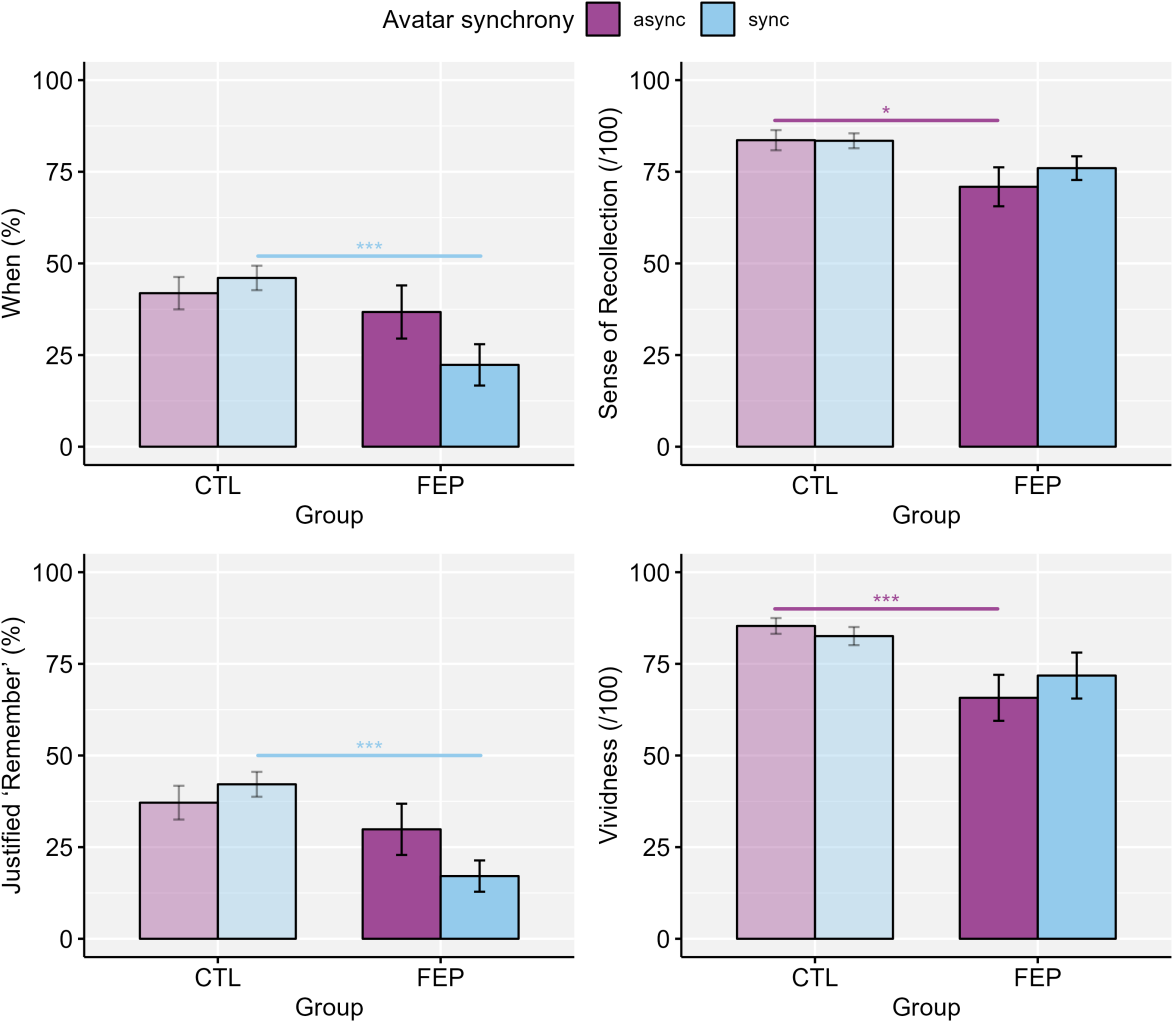
Significant interaction effect between Group and Avatar synchrony for temporal memory, sense of recollection, sense of remembering justified by correct contextual information, and memory vividness during retrieval. FEP, first-episode psychosis; CTL, healthy controls; Async., asynchronous avatar; Sync., synchronous avatar. Error bars represent the standard error (SE). *: p <.05; ***: p ≤.001.

The results of the robust ANOVA (**Table 5** and **Figure 6**) indicated that there were significant interaction effects of *Group* and *Avatar synchrony* on temporal episodic memory (‘When’), sense of recollection, sense of remembering justified by correct contextual information (Justified ‘Remember’), and memory vividness. Additionally, there were significant main effects of *Group* for ‘When’, Justified ‘Remember’, and Vividness, with lower scores for FEP than for CTL, irrespective of avatar synchrony. Follow-up analyses for the significant interactions indicated that in the synchronous avatar condition, FEP presented significantly lower scores than CTL for ‘When’ (*U* = 284.5, *p* = .001, *r_rb_* = 0.63) and Justified ‘Remember’ (*U =* 294.0, *p <*.001, *r_rb_* = 0.68), but there were no significant differences between groups for sense of recollection and vividness. In the asynchronous avatar condition, FEP presented significantly lower scores than CTL for sense of recollection (*U* = 256.0, *p* = .014, *r_rb_* = 0.46) and vividness (*U =* 285.0, *p = .*001, *r_rb_* = 0.63), but there were no significant differences between groups for ‘When’ and Justified ‘Remember’. There were no significant differences between avatar synchrony conditions across groups. Although the differences were not significant, in the CTL group, mean episodic memory scores for contextual information were slightly higher in the synchronous than the asynchronous condition.

**Table 5:** Episodic memory scores, by Group and Avatar synchrony.

	FEP		CTL		Robust ANOVA		
	Async.	Sync.	Async.	Sync.	Main effect of *Group*	Main effect of *Avatar synchrony*	Interaction effect
	*Mean (SD)*	*Mean (SD)*	*Mean (SD)*	*Mean (SD)*	*Q_a_, p*	*Q_b_, p*	*Q_ab_, p*
Contextual information							
What – Accuracy (%)	84.0 (18.4)	75.0 (10.8)	86.3 (13.7)	86.3 (15.0)	*Q_a_* = 7.13 *p* = .24	*Q_b_* = 1.14 *p* = .70	*Q_ab_* = -9.00 *p* = .12
What – Sensitivity (A’)	0.93 (0.09)	0.91 (0.07)	0.94 (0.05)	0.94 (0.06)	*Q_a_* = -0.01 *p* = .73	*Q_b_* = 0.005 *p* = .64	*Q_ab_* = -0.02 *p* = .19
Where (%)	69.6 (22.6)	69.9 (18.1)	77.1 (20.7)	82.7 (12.8)	*Q_a_* = 11.09 *p* = .15	*Q_b_* = -3.08 *p* = .36	*Q_ab_* = 4.72 *p* = .53
When (%)	36.7 (22.9)	22.3 (17.8)	41.9 (26.1)	46.0 (19.8)	***Q_a_* = 19.26** ***p* = .04**	*Q_b_* = -0.03 *p* = .996	***Q_ab_* = -26.27** ***p* = .04**
Source (%)	67.7 (23.3)	76.3 (17.0)	78.0 (18.9)	80.7 (16.7)	*Q_a_* = 8.09 *p* = .36	*Q_b_* = -4.02 *p* = .41	*Q_ab_* = 14.95 *p* = .14
Mean binding (/3)	1.06 (0.38)	0.92 (0.20)	1.19 (0.41)	1.28 (0.26)	*Q_a_* = 0.24 *p* = .053	*Q_b_* = -0.002 *p* = .99	*Q_ab_* = -0.24 *p* = .20
WWW Binding (%)	58.3 (19.6)	48.0 (8.49)	64.0 (18.8)	66.0 (14.6)	*Q_a_* = 12.23 *p* = .07	*Q_b_* = -0.23 *p* = .95	*Q_ab_* = -13.57 *p* = .07
Retrieval phenomenology							
Sense of Recollection (/100)	70.9 (16.8)	76.0 (10.2)	83.6 (16.3)	83.5 (12.1)	*Q_a_* = 9.67 *p* = .051	*Q_b_* = 0.58 *p* = .80	***Q_ab_* = 7.92** ***p* = .048**
Justified ‘Remember’ (%)	29.9 (22.1)	17.1 (13.5)	37.1 (27.3)	42.1 (20.1)	***Q_a_* = 22.5** ***p* = .04**	*Q_b_* = -4.02 *p* = .50	***Q_ab_* = -25.27** ***p* = .04**
Perspective (/100)	65.9 (25.1)	63.2 (32.7)	76.6 (22.4)	75.5 (21.1)	*Q_a_* = 6.49 *p* = .58	*Q_b_* = 0.08 *p* = .96	*Q_ab_* = -1.61 *p* = .88
Vividness (/100)	65.7 (19.8)	71.8 (19.8)	85.3 (12.7)	82.6 (14.6)	***Q_a_* = 17.08** ***p* = .04**	*Q_b_* = 0.90 *p* = .53	***Q_ab_* = 11.74** ***p* = .03**
Fidelity (/100)	68.3 (14.3)	74.8 (12.4)	79.1 (14.7)	76.9 (15.1)	*Q_a_* = 4.91 *p* = .40	*Q_b_* = -0.74 *p* = .55	*Q_ab_* = 2.61 *p* = .37
Emotional Intensity (/100)	52.9 (15.8)	51.4 (20.6)	58.7 (20.1)	56.9 (20.1)	*Q_a_* = 5.63 *p* = .46	*Q_b_* = 1.64 *p* = .15	*Q_ab_* = -1.94 *p* = .58
Self-Reference (/100)	38.2 (16.7)	38.3 (20.6)	42.7 (20.2)	42.7 (20.3)	*Q_a_* = 6.50 *p* = .36	*Q_b_* = -1.92 *p* = .38	*Q_ab_* = -2.45 *p* = .59

FEP, first-episode psychosis; CTL, healthy controls; Async., asynchronous avatar; Sync., synchronous avatar; Q, robust ANOVA statistic; p, robust ANOVA p-value. In bold: statistically significant results.

### 3.5 Relationships between the sense of self, sense of embodiment, episodic memory, and psychopathology

**Figure 7:**
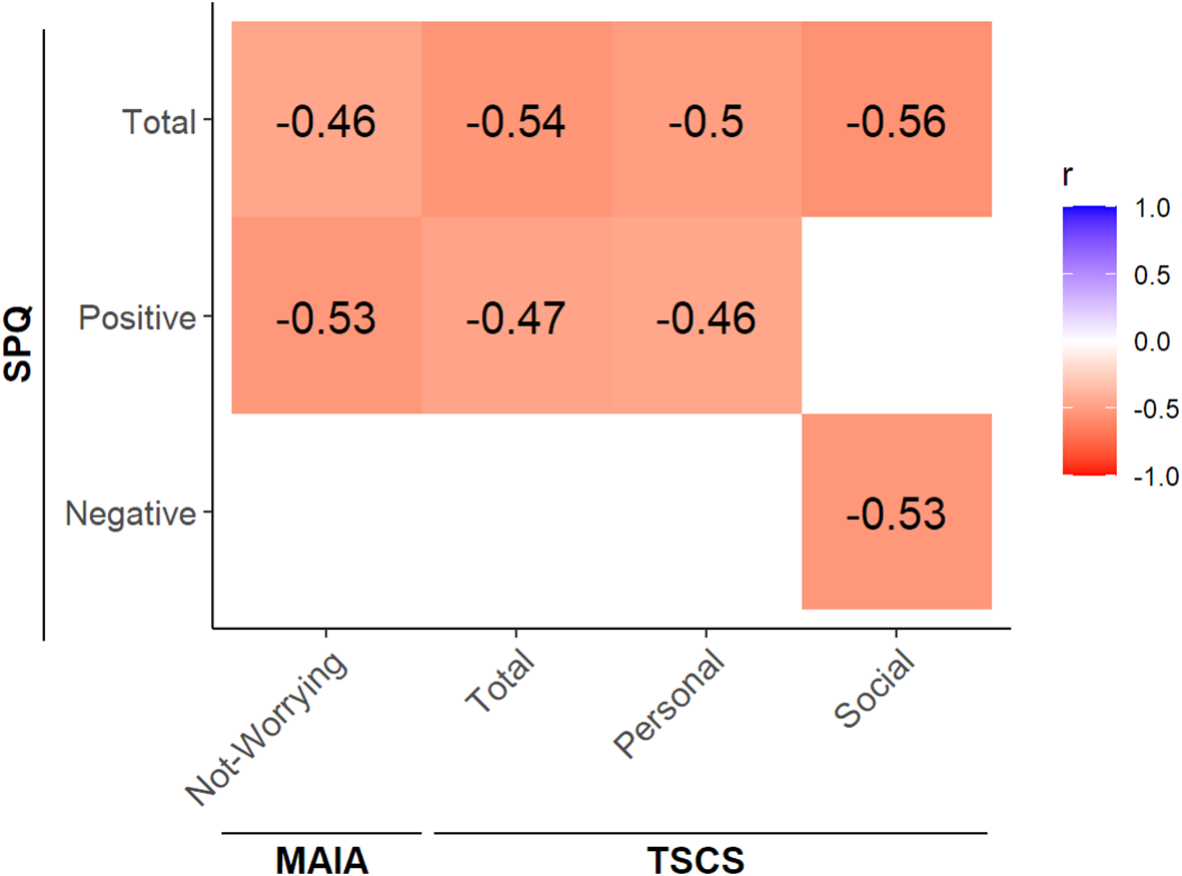
Significant correlations between schizotypal traits and the sense of minimal Self and narrative Self for the CTL group. CTL, healthy controls; SPQ, Schizotypal Personality Questionnaire; MAIA, Multidimensional Assessment of Interoceptive Awareness – Version 2; TSCS, Tennessee Self-Concept Scale – Second Edition, Short Form. All correlations are significant at α = 0.05 significance level, after adjusting for multiple comparisons using Benjamini-Hochberg correction.

Correlational analyses between psychopathology and the sense of Self did not reveal any significant correlations in the FEP group. For CTL, there were significant correlations between schizotypal traits and self-reported sense of Self (**Figure 7**). Overall, the correlations indicated that greater severity of schizotypal traits was associated with an increased tendency to worry and to experience emotional distress due to bodily sensations of pain or discomfort (MAIA Not-Worrying), and with a poorer sense of narrative Self, especially a poorer self-esteem (TSCS Total), and poorer personal and social representations of the Self.

Correlational analyses between the sense of embodiment and the sense of Self revealed that for FEP, enhanced sense of minimal Self was associated with a stronger sense of embodiment. In particular, the experience of one’s body as safe and trustworthy (MAIA Trust) was found to be strongly and significantly associated with a stronger global sense of embodiment over both the synchronous and the asynchronous avatar (*r* = 0.86, *p* = .002, *p_adj_* = .044; *r* = 0.91, *p* = .002, *p_adj_* = .018, respectively), as well as a higher sense of self-location with the synchronous avatar (*r* = 0.83, *p* = .003, *p_adj_* = .049). For CTL, enhanced sense of minimal Self was also associated with a stronger sense of embodiment, although less strongly than for FEP. In particular, a heightened awareness of the interconnection between bodily sensations and emotional states (MAIA Emotional Awareness) in CTL was significantly correlated with a higher sense of self-location with the synchronous avatar (*r* = 0.49, *p* = .003, *p_adj_* = .037).

Correlational analyses between the sense of embodiment and psychopathology did not reveal any significant correlations in the FEP group. For CTL, SPQ Total and Positive scores were significantly positively correlated with BIQ Self-location in the synchronous avatar condition (*r =* 0.58, *p* <.001, *p_adj_* = .002; *r =* 0.58, *p* <.001, *p_adj_* = .002, respectively).

Correlational analyses between episodic memory and psychopathology indicated that for FEP, greater severity of neurological soft signs, in particular sensorimotor integration, was associated with worse episodic memory, although the correlations did not remain significant after correction for multiple comparisons. In the synchronous avatar condition, NSSE Motor Integration factor was negatively correlated with memory Binding (*r* = -0.70, *p* = .036, *p_adj_* = .427), and NSSE Sensory Integration factor was negatively correlated with Accuracy (*r* = -0.83, *p* = .010, *p_adj_* = .202), Sensitivity (*r* = -0.81, *p* = .015, *p_adj_* = .241), and Binding (*r* = -0.73, *p* = .041, *p_adj_* = .427). In the asynchronous avatar condition, NSSE Total score was negatively correlated with Sensitivity (*r* = -0.71, *p* = .048, *p_adj_* = .308), NSSE Motor Integration factor was negatively correlated with Source Memory (*r* = -0.74, *p* = .021, *p_adj_* = .228), and NSSE Sensory Integration factor was negatively correlated with Accuracy (*r* = -0.73, *p* = .040, *p_adj_* = .292) and Sensitivity (*r* = -0.83, *p* = .011, *p_adj_* = .173). For CTL, in the asynchronous avatar condition, SPQ Positive was negatively correlated with Temporal Memory (*r* = -0.45, *p* = .01, *p_adj_* = .03), Justified ‘Remember’ (*r* = -0.42, *p* = .01, *p_adj_* = .05), and Mean Binding (*r* = -0.51, *p* = .002, *p_adj_* = .01).

## 4 Discussion

In this pilot study, we successfully induced a stronger sense of embodiment over a virtual avatar using synchronous compared to asynchronous visuomotor stimulation in FEP patients, similar to CTL. However, we found that this strong embodiment condition was associated with an altered spatial representation of the body in the virtual space for FEP patients. We highlighted the specific impact of embodiment during incidental encoding on subsequent episodic memory for naturalistic events. For naturalistic events encoded in the strong embodiment condition, FEP patients performed significantly worse than CTL for contextual information recognition, but their ratings were comparable to those of CTL for retrieval phenomenology. Conversely, for events encoded in the weak embodiment condition, FEP performed comparably to CTL for contextual information recognition, but rated their retrieval phenomenology significantly lower than CTL. Overall, the CTL did not demonstrate statistically significant differences in recognition memory performance between the strong and weak embodiment conditions, although the mean scores for contextual information were slightly higher in the strong embodiment condition.

### 4.1 Full-body illusion and sense of embodiment

The full-body illusion based on synchronous visuomotor stimulation successfully induced a strong sense of embodiment over a generic gender-matched avatar embodied from a first-person perspective in FEP patients. Moreover, patients exhibited similar susceptibility to the full-body illusion as healthy participants. This finding is consistent with the results of a recent systematic review of full-body illusions in the schizophrenia spectrum, which showed that patients on the schizophrenia spectrum presented a higher susceptibility to the full-body illusion than healthy people in paradigms using visuotactile stimulation, but not in paradigms based on visuomotor stimulation, where patients showed the same level of embodiment as healthy participants (101). Additionally, results from the mental imagery task, which assessed the perceived space occupation by the virtual body via the distance to a ball, indicated that CTL stopped the ball at a greater distance from them in the synchronous condition than in the asynchronous condition, reflecting a greater occupation of space by the virtual body in the strong embodiment condition. This result is consistent with that of another study, which showed that participants stopped further away from the boundaries of the zone when navigating with a virtual body than when navigating without a seen body, reflecting a drift in their self-location (102, 103). Conversely, this behaviour was not observed in FEP patients, as their distance to the ball was reduced in the strong embodiment condition. This may indicate an alteration in their representation of their body in virtual space, which could reflect their minimal self-disorders. Moreover, this finding of a reduced occupation of virtual space by the body in FEP patients is consistent with accounts of a narrower and shallower peripersonal space in schizophrenia, as demonstrated in both real-life (17, 104, 105) and virtual reality settings (106).

Although the overall sense of embodiment, as assessed by a self-reported questionnaire, was significantly stronger with the synchronous than with the asynchronous avatar for both groups, no significant differences were observed between these two conditions for the individual subcomponents of embodiment. This may be attributed to inter-individual differences in susceptibility to the full-body illusion, implying that larger sample sizes are necessary to observe significant differences. Indeed, several studies have reported ‘non-responders’ to the rubber hand illusion (107), which is the princeps paradigm from which the full-body illusion was derived. The present study provides new insights into the potential mechanisms underlying this inter-individual variability.

In FEP patients, a higher sense of minimal Self, especially a stronger experience of one’s body as safe and trustworthy, was correlated with a stronger global sense of embodiment over both the synchronous and the asynchronous avatar. This is consistent with the proposition that schizophrenia patients rely less on stored body representations and more on online multisensory input due to unreliable internal predictions, resulting in an ‘over-inclusive’ agency (101, 108). This ‘over-inclusive’ agency has been associated with reduced self-demarcation (101), i.e. greater difficulty in delineating the Self from the external world. Our findings lend support to this proposition, as self-demarcation relies on peripersonal space and the representation of the Self in space, yet we found that stronger sense of embodiment was associated with an altered spatial representation of the body in FEP patients, which would consequently impact self-demarcation.

In the healthy population, people who demonstrated a heightened awareness of the interconnection between bodily sensations and emotional states exhibited a greater susceptibility to the illusion with a synchronous avatar, suggesting that boosting emotional awareness may be an effective approach to increasing susceptibility to the full-body illusion. For instance, emotional awareness could be enhanced by providing visual feedback of breathing rate or heart rate when people are in different emotional states, thereby facilitating their awareness of the connection between interoceptive bodily sensations and emotional states. Cardio-visual feedback has been used to induce the rubber hand illusion: synchronous cardio-visual feedback successfully induced a strong sense of ownership (109).

### 4.2 Embodiment and episodic memory in the healthy population

In healthy participants, the strong embodiment condition did not significantly enhance episodic memory for naturalistic events, as assessed via a recognition task. Although mean scores for contextual information were higher for events encoded with a synchronous avatar compared to an asynchronous avatar, this difference was not statistically significant. This may be attributed to the nature of the episodic memory assessment. Studies have shown that recognition tasks tend to be less challenging than free recall tasks (110), and are therefore often less sensitive in detecting differences in memory performance. The recognition task used in the present study might have been insufficiently challenging for healthy young participants due to the relatively low number of events to encode and the short retention interval between encoding and testing. These factors likely contributed to a ceiling effect, where high memory performance was observed in both experimental conditions. Indeed, in a preceding study in healthy young participants that inspired the current research (45), 36 events were included for encoding, compared to 20 in the current study, due to the addition of a third experimental condition (in which no body was visible in the encoding scene) and two additional events per condition. Furthermore, memory was assessed both immediately and after a ten-day delay, using both free recall and recognition tasks. The population in that earlier study shared comparable sociodemographic characteristics with the CTL group in the present study (38 young adults, mean age of 22.1 years, 70% female). Despite the more challenging design in the earlier study, only one significant difference between the asynchronous and synchronous avatar conditions emerged in the immediate recognition test, namely the number of correctly recognised events (i.e., ‘hit’ rate). However, significant differences were observed between avatar synchrony conditions in the delayed recognition task (e.g., false alarm rate, sensitivity), and in the free recall test at both delays (e.g., number of recalled events, specific details, binding), indicating that the effect of the embodiment condition on the episodic memory of naturalistic events was genuine, but could only be detected using more sensitive memory measures, such as free recall or delayed recognition. The simplifications in the current study were implemented to reduce the overall duration of the experimental session, thus alleviating the burden on the FEP patients. However, these modifications likely reduced the sensitivity of the memory assessment for the CTL group.

### 4.3 Embodiment and impairment of contextual aspects of episodic memory in early psychosis

The strong embodiment condition was associated with impaired contextual aspects of episodic memory in FEP, in particular poorer temporal episodic memory and a lower sense of remembering justified by correct contextual information. While FEP patients had comparable scores to CTL in recognising contextual information for events encoded with an asynchronous avatar, they performed significantly worse than CTL for events encoded with a synchronous avatar. This suggests that FEP patients may have struggled to rely on a coherent, synchronous representation of their minimal Self to effectively encode naturalistic events into strong episodic memory traces. Indeed, episodic memory formation relies on coherent multisensory integration anchored to the body, which defines an egocentric reference frame that can be used to bind contextual details into strong memory traces in the hippocampus, that can then be reinstated during the retrieval of events (53, 54, 111). Impairments in the spatial representation of the body in healthy individuals have been shown to result in altered episodic memory (53). In our study, the episodic memory deficits observed in FEP patients may, therefore, be related to the altered spatial representation of the body observed in the synchronous avatar condition. Indeed, the minimal self-disorders experienced by schizophrenia patients include alterations in their body representation (112), which are associated with multisensory integration deficits (113). It can thus be posited that the deterioration of the episodic memory of naturalistic events in FEP patients may be attributable to impairments in multisensory integration and in the spatial representation of the patients’ bodies. This could be corroborated by our findings that more severe sensorimotor integration deficits, as assessed by the NSSE, correlated with worse episodic memory accuracy, sensitivity, and binding for events encoded with both a synchronous and asynchronous avatar. Although these correlations did not remain significant after correcting for multiple comparisons, they were strong and, if confirmed in future studies with a larger sample size, they may offer valuable insights into the relationship between minimal self-disorders, multisensory integration deficits, and episodic memory impairments in FEP patients.

Prior research has demonstrated that chronic schizophrenia patients presented a critical impairment in the conscious recollection and binding of episodic memory and episodic autobiographical memory (30, 114). Our results extend those previous findings by showing that the sense of remembering is already impaired in early stages of psychosis, and that this deficit could be attributable to disorders of the minimal Self. This has significant implications, as conscious recollection is a prerequisite for the formation and consolidation of autobiographical memories, which in turn ensure the phenomenological continuity of the subjective sense of Self (1). Therefore, our results highlight the bidirectional relationship between self-disorders and episodic memory deficits in the schizophrenia spectrum. Altogether, our findings indicate that interventions targeting disorders of the minimal Self may prove beneficial in improving episodic memory across the schizophrenia spectrum. This is of particular significance given that episodic memory impairments in schizophrenia have been associated with poor functional outcomes and reduced quality of life (30, 115, 116), hence the potential benefit of treating episodic memory impairment on patients’ functional outcomes (117).

### 4.4 Embodiment and phenomenology of episodic memory retrieval in early psychosis

The strong embodiment condition was associated with impaired phenomenology of episodic memory retrieval in FEP, in particular an altered sense of recollection and memory vividness. Indeed, FEP patients rated their sense of recollection and memory vividness lower than CTL for the events encoded with an asynchronous avatar, but not for the events encoded with a synchronous avatar, even though their memories were objectively less accurate in the synchronous but not in the asynchronous avatar condition. Notably, in the strong embodiment condition, patients rated their sense of recollection similarly to the CTL group, but when this self-rated score was adjusted using the objective memory binding score, their sense of remembering justified by correct contextual information was significantly lower than that of the CTL group. This finding may reflect a possible alteration in the meta-cognitive processes involved in assessing subjective memory quality, i.e. a meta-memory impairment. Indeed, it aligns with previous studies that have demonstrated an overconfidence in false memories in schizophrenia (118, 119). This bias is associated with knowledge corruption, leading patients to be overly confident in accepting incomplete information as satisfactory during decision-making processes (118, 119). More broadly, meta-memory impairment in schizophrenia is associated with altered source monitoring, which encompasses the processes involved in attributing the origin of subjective experiences (119, 120). In light of these findings, it can be posited that the altered spatial representation of the body observed in the synchronous condition is associated with impairments in meta-memory and source monitoring in FEP patients. This would be consistent with the hypothesis that psychosis is particularly characterised by internal source monitoring deficits, which are associated with reduced self-demarcation and unreliable internal predictions (121). In line with the meta-analysis conducted by Damiani and colleagues (121), which showed specific alterations in internal source monitoring but no deficits in other source monitoring subtypes, our results did not reveal deficits in external source monitoring in FEP patients. Our results also indicate that disorders of the minimal Self may be a root cause of metacognitive deficits and poor insight in schizophrenia, as has been proposed in the literature (122). Therefore, targeting disorders of the minimal Self may also improve insight in the schizophrenia spectrum, which may be beneficial, as poor insight has been associated with a number of adverse outcomes in schizophrenia, including poor medication adherence (123) and altered psychosocial functioning (124, 125).

### 4.5 Influence of schizotypal personality traits on sense of self, embodiment, and episodic memory

Our results indicated that higher levels of schizotypy were correlated with a lower sense of minimal Self and narrative Self in CTL, specifically an increased tendency to worry or experience emotional distress with sensations of pain or discomfort and lower self-esteem. These findings align with previous research that has demonstrated a correlation between self-disorders and higher levels of schizotypy (25, 27, 126). Peripersonal space has also been shown to be altered in schizotypy (127). Our findings lend further support to the hypothesis that self-disorders are specific to the schizophrenia spectrum and are already present in early, subclinical stages of the pathology.

Higher levels of schizotypy, particularly positive schizotypy, were also correlated with a stronger sense of embodiment of the synchronous avatar, which has also been observed in studies using the rubber hand illusion (18, 128). Therefore, the present study extends these previous findings to the full-body illusion. Additionally, higher levels of positive schizotypy were correlated with reduced episodic memory scores (temporal memory, binding, and justified ‘Remember’), suggesting generalised episodic memory deficits in schizotypy, again consistent with the literature (129, 130), and supporting the fully dimensional model of schizotypy, with parallel findings between schizotypal personality in the healthy population and schizophrenia (28, 131).

### 4.6 Strengths and limitations of the study

To the best of our knowledge, the full-body illusion using immersive virtual reality has not previously been studied in the FEP, and even less so the interplay between the minimal Self and the episodic memory of naturalistic events. Our findings indicated that alterations in the minimal Self in the FEP may lead to impaired episodic memory and meta-memory. They also highlighted that targeting disorders of the minimal Self may have many benefits in terms of quality of life, psychosocial outcomes, and treatment adherence across the schizophrenia spectrum, by improving episodic memory and insight. We therefore support the idea of developing a phenomenological psychotherapy for schizophrenia (132), for instance through body-oriented psychotherapy, which has demonstrated efficacy in alleviating negative symptoms in schizophrenia (133, 134). Furthermore, the investigation of schizotypal personality traits in healthy participants has provided further support for the fully dimensional model of schizotypy, with parallels between schizotypal personality in the healthy population and schizophrenia (28, 126, 131).

Nevertheless, several limitations must be considered. First, the small sample size of FEP patients limits the generalisability of the results and the pilot study must be extended. As Nelson has pointed out (29), “not all first-episode psychosis is the same”, emphasising on the need for larger sample sizes to account for the high inter-individual variability. This is particularly important given that our study focuses on the comprehensive episodic memory of complex events, for which subtle assessment is prone to greater inter-individual variability than the memory of simplistic words or objects.

Second, regarding our experimental procedure, we observed that the duration of navigation was significantly longer in the asynchronous compared to the synchronous avatar condition for the CTL group. This could be another explanation for the lack of significant differences in memory performance between the two embodiment conditions, suggesting that participants may have compensated for the altered encoding conditions induced by the asynchronous avatar by spending more time fixating on events. Longer navigation duration may also reflect a higher cognitive load in the asynchronous avatar condition, which future studies could monitor using tools such as the NASA Task Load Index (135). In our future studies, the use of virtual reality head-mounted displays with integrated eye-tracking technology should facilitate the control of the number and duration of fixations on each event, providing an objective measure of attention and engagement during memory encoding.

Third, the variability between the naturalistic events used for memory encoding (e.g., differences in event duration, valence, or complexity) may represent a limitation of the current study. Nevertheless, these events have been demonstrated to enable the investigation of naturalistic memory and its associations with the Self in situations that closely resemble everyday life, with a strong sense of presence and over long-term retention periods (45). This investigation of naturalistic memory was the primary focus of our study, rather than examining memory for standardised experimental material. Moreover, the counterbalanced design of the current study enabled the minimisation of potential systematic bias associated with specific event characteristics, thereby minimising the likelihood that differences in event characteristics affected overall memory performance across conditions. Future research would still benefit from a formal characterisation of the naturalistic events to ensure a more balanced allocation of events across conditions. Again, integrating eye-tracking should provide valuable insights to address the potential impact of event variability.

Fourth, the results indicated that alterations in body representation and multisensory integration may play a crucial role in the episodic memory deficits observed in the FEP. To support this assertion, it would be relevant to include in future studies a task that measures the peripersonal space, which is characterised by facilitated multisensory integration (9, 10). Such tasks have already been developed based on multisensory integration and reaction time, using audio-tactile (10, 136) or visuo-tactile stimulation (106, 137), in both real-life and virtual reality settings. One study has shown that, despite alterations in peripersonal space in schizophrenia, its plasticity was preserved, and the authors were able to induce an extended and steeper peripersonal space through a tool-use motor training (105). Therefore, if it is confirmed that the altered body representation and peripersonal space are involved in the impaired episodic memory in the schizophrenia spectrum, then using such motor training to restore the peripersonal space may also lead to improved episodic memory in patients across the schizophrenia spectrum.

## 5 Conclusion

Disturbances of the minimal Self in the first-episode psychosis are associated with impaired episodic memory, which, in turn, affects patients’ quality of life and psychosocial outcomes. These findings highlight the necessity of targeting disorders of the minimal Self in the treatment of schizophrenia spectrum disorders, as well as the importance of early intervention programmes. Additionally, the findings support the fully dimensional model of schizotypy, indicating parallels between schizotypal traits in the healthy population and schizophrenia. A subsequent study will be conducted to clarify the interrelationships between the multifaceted Self and episodic memory in the schizophrenia spectrum, addressing the limitations observed in the pilot study and investigating the individual and joint influence of both minimal and narrative facets of the Self on episodic memory.

## Data Availability

The raw data supporting the conclusions of this article will be made available by the corresponding authors, without undue reservation.
