# Supplementary material for: Impact of minimal self disorders on naturalistic episodic memory in first-episode psychosis and parallels in healthy individuals with schizotypal traits
Source: Front Psychiatry. 2024 Nov 13;15:1469390. doi: 10.3389/fpsyt.2024.1469390 (PMC11598521; doi:10.3389/fpsyt.2024.1469390)
Supplement: Supplementary file 1 [file Table1.pdf]

Supplementary Material

Supplementary Table S1. Description and illustration of the naturalistic events used for episodic memory encoding.

Event number	Event description	Event illustration
Event A1	A homeless man hails the passers-by and begs for money	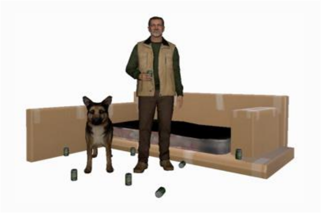
Event A2	A man calls out to passers-by to watch out for three hens on the road that have escaped from a poultry house	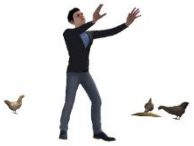
Event A3	A litter bin catches fire	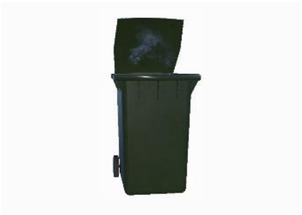
Event A4	An angry man throws a stone at a car and smashes its windscreen	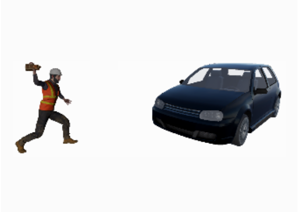
Event A5	Two men take a yoga class with a female coach	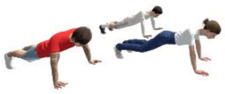
Event A6	A man plays a Latin tune on his guitar	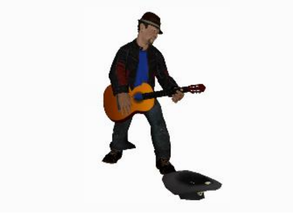

Event A7	An impatient woman overtakes four other customers in a market queue	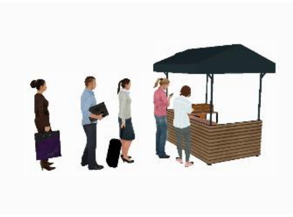
Event A8	Two men dance hip-hop to rap music	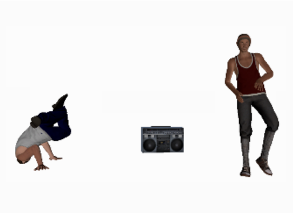
Event A9	Two women are waiting for the bus at a bus shelter and complain that the timetable display screen has broken down again	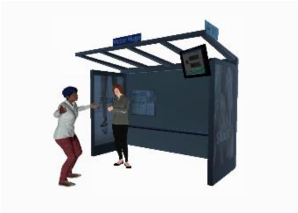
Event A10	A woman feeds and pets a cat	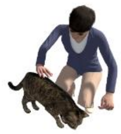
Event B1	Numerous 100-euro banknotes escape from a broken-down ATM	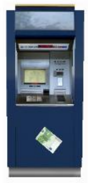
Event B2	A dog barks aggressively at passers-by	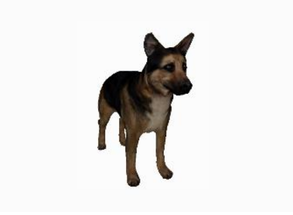
Event B3	A man driving a luxury car gets angry and honks his horn because the road is blocked by barriers	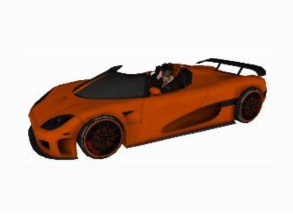

Event B4	A couple kissing	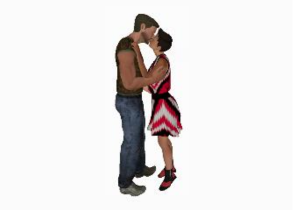
Event B5	A clumsy customer spills a bottle of wine on the waitress terrace of a restaurant and apologises	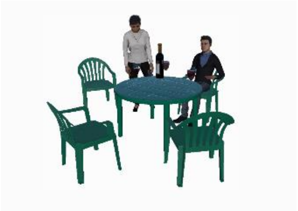
Event B6	A man takes photographs of a woman dancing	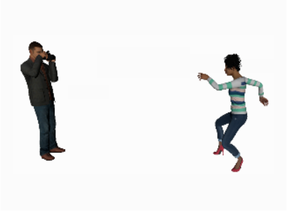
Event B7	An elderly woman trips over a stone, spilling her groceries on the ground	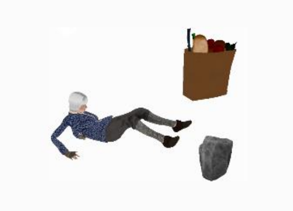
Event B8	A woman laments the loss of her bakery, which is on fire	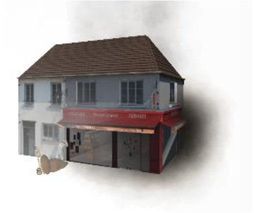
Event B9	A couple gets excited about three rabbits and suggests adopting one	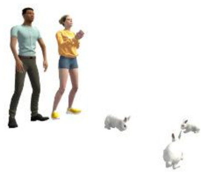
Event B10	A man plays a classical tune on the piano	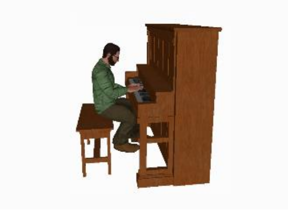

Supplementary Video S1. Excerpts of the embodiment procedure using synchronous or asynchronous visuomotor stimulation in front of a virtual mirror.

File *Yeh2024FrontiersMovieS1.mp4* on the OSF project:
https://osf.io/ctzme/?view_only=41ee457049594f218893f46c0ec3f5e5

Supplementary Video S2. Excerpts from the incidental encoding of naturalistic events while navigating with a synchronous or asynchronous avatar in the virtual city.

File *Yeh2024FrontiersMovieS2.mp4* on the OSF project:
https://osf.io/ctzme/?view_only=41ee457049594f218893f46c0ec3f5e5
